# Balance Assessments Using Smartphone Sensor Systems and a Clinician-Led Modified BESS Test in Soccer Athletes with Hip-Related Pain: An Exploratory Cross-Sectional Study

**DOI:** 10.3390/s26031061

**Published:** 2026-02-06

**Authors:** Alexander Puyol, Matthew King, Charlotte Ganderton, Shuwen Hu, Oren Tirosh

**Affiliations:** 1 School of Health and Biomedical Sciences, RMIT University, Bundoora, VIC 3083, Australia; s4108405@student.rmit.edu.au (A.P.); charlotte.ganderton@rmit.edu.au (C.G.); 2 School of Allied Health, Human Services and Sport, La Trobe University, Plenty Road, Bundoora, VIC 3086, Australia; m.king@latrobe.edu.au; 3 School of Science, RMIT University, Melbourne, VIC 3000, Australia; shuwen.hu@rmit.edu.au

**Keywords:** hip pain, balance, smartphone, remote assessment, BESS test

## Abstract

Background: The Balance Error Scoring System (BESS) is the most practiced static postural balance assessment tool, which relies on visual observation, and has been adopted as the gold standard in the clinic and field. However, the BESS can lead to missed and inaccurate diagnoses—because of its low inter-rater reliability and limited sensitivity—by missing subtle balance deficits, particularly in the athletic population. Smartphone technology using motion sensors may act as an alternative option for providing quantitative feedback to healthcare clinicians when performing balance assessments. The primary aim of this study was to explore the discriminative validity of an alternative novel smartphone-based cloud system to measure balance remotely in soccer athletes with and without hip pain. Methods: This is an exploratory cross-sectional study. A total of 64 Australian soccer athletes (128 hips, 28% females) between 18 and 40 years completed single and tandem stance balance tests that were scored using the modified BESS test and quantified using the smartphone device attached to their lower back. An Exploratory Factor Analysis (EFA) and a Clustered Receiver Operating Characteristic (ROC) using an Area Under the Curve (AUC) were used to explore the discriminative validity between the smartphone sensor system and the modified BESS test. A Linear Mixed-Effects Analysis of Covariance (ANCOVA) was used to determine any statistical differences in static balance measures between individuals with and without hip-related pain. Results: EFA revealed that the first factor primarily captured variance related to smartphone measurements, while the second factor was associated with modified BESS test scores. The ROC and the AUC showed that the smartphone sway measurements in the anterior–posterior and mediolateral directions during single-leg stance had an acceptable to excellent level of accuracy in distinguishing between individuals with and without hip-related pain (AUC = 0.72–0.80). Linear Mixed-Effects ANCOVA analysis found that individuals with hip-related pain had significantly less single-leg balance variability and magnitude in the anteroposterior and mediolateral directions compared to individuals without hip-related pain (*p* < 0.05). Conclusion: Due to the ability of smartphone technology to discriminate between individuals with and without hip-related pain during single-leg static balance tasks, it is recommended to use the technology in addition to the modified BESS test to optimise a clinician-led assessment and to further guide clinical balance decision-making. While the study supports smartphone technology as a method to assess static balance, its use in measuring balance during dynamic movements needs further research.

## 1. Introduction

Postural static balance assessments can be used to assess an athlete’s performance and return to play following injury. For example, the assessment of static balance is recommended in clinical practice guidelines for athletes with chronic ankle instability (CAI) [1,2], and is recommended in the Sport Concussion Assessment Tool 6 (SCAT6) protocol for concussion management, diagnosis, and readiness to return to sport [3]. It has been reported that altered postural balance is seen in soccer players with groin pain [4] and in individuals with refractory greater trochanteric pain syndrome [5]. Hip-related pain, which is common in soccer athletes and affects between 45 and 59% of players during a competitive season [6,7,8], may impede static balance and the performance of these skills, due to altered neuromuscular control [9,10] and altered hip and pelvic biomechanics seen in symptomatic individuals [11,12,13,14,15].

The Balance Error Scoring System (BESS) is a commonly used and recommended assessment tool in clinical settings, which relies on visual subjective observation from a clinician [16]. Although the BESS test was initially developed to evaluate concussion [17], its utility as an assessment tool has been further evaluated in lower limb musculoskeletal conditions, where balance and proprioception are impaired [18], including in osteoarthritis [19], CAI [16], and more recently, hip pain [10]. This research has now transitioned into exploring the BESS test in lower limb injury risk [20]. Although used in lower limb musculoskeletal conditions, the visual assessment has limitations. For example, visual balance assessments have poor sensitivity (34–64%) and discriminative ability to detect meaningful clinical improvements or changes in balance in individuals with concussion and hip-related pain [10,21,22,23,24], which may be reflected by both the subjective nature and reliance on individual clinical expertise for such tests [22]. Furthermore, whilst the BESS test has previously shown to be able to detect balance impairments in individuals with CAI, it has been unable to substantiate these differences in comparison with controls [16].

Smartphone technology utilising the accelerometer (linear acceleration) and gyroscope (angular velocity) motion sensors is one valid and reliable tool that can detect changes in two dimensions, including the anterior–posterior (forward–backwards) and mediolateral (sideways) postural sway during static, dynamic, and functional balance tasks in young and older community-dwelling adults [22,25,26,27,28]. Several computer-based systems and applications using smartphone sensors have been designed to detect changes in postural trunk sway in the frontal and sagittal planes [23,26,29]. Not only do these tools have a clinical advantage in being both user-friendly and cost-effective, but they have also been validated against gold-standard apparatuses such as force plates [22] and laboratory motion capture systems [30]. One such design is the TelePhysio (version 1.0) [23] application, which allows individuals to connect to a clinician remotely through a web-based cloud system via their own smartphone device [23,27]. Similarly to other smartphone applications [22], TelePhysio [23] measures the magnitude and variability of postural sway in both the mediolateral and anterior–posterior directions. Magnitude is defined as the amount of overall sway in a certain direction, and variability refers to the change in sway away from the mean [22].

Previous studies [22,23,26,29] have demonstrated the efficacy of smartphone sensors in measuring static and dynamic balance in individuals with concussion, stroke, and chronic ankle instability. However, to the authors’ knowledge, only one study has used this technology to assess dynamic balance during repeated squat tasks in individuals with and without hip-related pain [27]. Although this study explored fundamental dynamic balance differences between symptomatic and asymptomatic groups [27], it did not compare the smartphone with the modified BESS test and did not explore the ability of smartphone sensors to detect whether an individual had hip-related pain. Moreover, there have been no studies examining static balance using a smartphone sensor system in an athletic population. This is furthered by the lack of studies exploring the deterministic qualities across static balance tasks in individuals with and without hip-related pain. This study has been designed to fill this evidence gap, given the uncertainty surrounding the BESS test and smartphone systems in the athletic population, and to examine whether altered static balance is demonstrated in individuals with hip-related pain.

Therefore, the primary aim of this study is to determine the discriminative validity of the smartphone sensor system using the TelePhysio application in contrast with the modified BESS test to detect changes in static balance measures, and to assess whether these balance measures differentiate between soccer athletes with and without hip-related pain. The secondary aim of this study is to compare the differences in static balance in male and female soccer athletes with and without hip-related pain.

## 2. Materials and Methods

**Figure 1:**
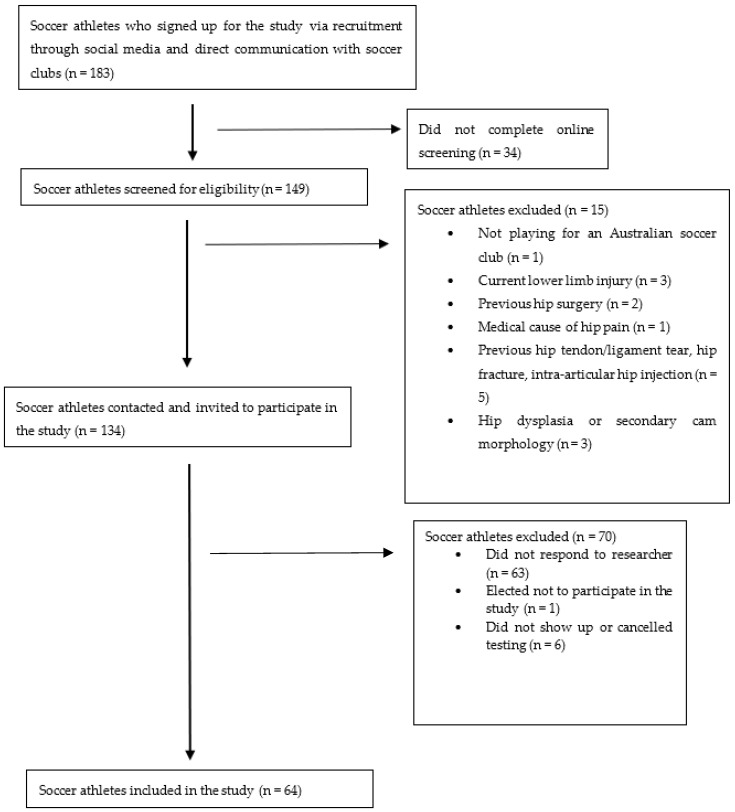
Diagram outlining the participant selection process.

This exploratory cross-sectional study adhered to *the Strengthening the Reporting of Observational studies in Epidemiology (STROBE)* guidelines [31]. Ethics approval was granted by the University Human Ethics Committee (ref: 2024-27894-25352) and aligned with the Declaration of Helsinki. Participants were recruited via direct communication with Australian soccer clubs and through social media (i.e., Instagram, Facebook, and LinkedIn). Participants were then directed to fill out a participant information and consent form, followed by a specific inclusion and exclusion screening questionnaire to assess their eligibility for the study. Participants were then contacted by the researcher to gain further verbal consent and to organise testing (Figure 1).

### 2.1. Participants

Sixty-four (128 hips) soccer athletes (females: 18, 28%) between 18 and 40 years were recruited as part of a larger longitudinal study. Participants were excluded if they reported the following: a previous ankle injury within the past two weeks; a previous hip surgery (i.e., arthroscopy or ORIF) or a planned hip surgery in the next 12 months; a previous hip tendon avulsion(s), hip tendon tear(s), hip ligament rupture(s) or pelvic/hip fracture(s); an intra-articular hip–joint injection(s) in the past 3 months; a medical cause of hip pain (autoimmune, neurological or oncological); hip dysplasia or a secondary CAM morphology (i.e., resulting from previous Perthes disease, slipped capital epiphysis, or acetabular dysplasia) on radiograph; and/or inability to understand the English language. The exclusion of individuals with a medical cause of hip pain or a planned hip surgery was imposed to ensure that (1) individuals with hip-related pain remained classified as non-surgical participants, and (2) to limit the wide-ranging and potentially different symptoms that individuals with a medical cause of hip pain may have had. Participants were deemed to have hip-related pain if they reported hip-related (anterior hip, groin, or thigh) pain that was aggravated by prolonged sitting or kicking, with an intensity of ≥3/10 on a numerical pain scale. All participants who were tested face-to-face by the researcher, and who reported hip-related pain, underwent a clinical examination (FADIR test) to determine whether their pain was of an intra-articular or extra-articular origin [32]. All participants provided informed written and verbal consent before testing.

### 2.2. Hip Pain Measurement

Prior to balance testing, each participant completed the International Hip Outcome Tool (iHOT-33) outcome measure. The iHOT-33 is a participant self-reported questionnaire that evaluates symptoms and functional limitations, sport and recreational activities, as well as job-related, social, emotional, and lifestyle-related concerns [15,33]. It has shown to have acceptable construct, content, and discriminative validity in non-surgical young and middle-aged individuals with hip and groin pain at a group level [33].

### 2.3. Balance Measurement

**Figure 2:**
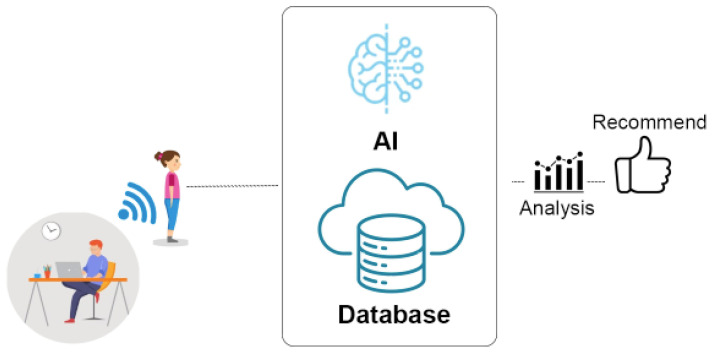
The TelePhysio application used to connect the researcher to the participant.

Individuals with and without hip-related pain were measured on one occasion simultaneously using the modified BESS test scoring protocol and the smartphone TelePhysio (Version 1) application [23]. The technology utilises smartphone motion sensors with Wi-Fi capability, allowing researchers to perform remote balance tele-assessments. With the smartphone device, researchers connect to participants online through a web-browser-based platform and control the motion-sensor data capture (Figure 2). This remote connection allows the researchers to attain the acceleration and angular velocity data through the smartphone motion sensors in real time. Participants attached their own smartphone device to a belt, which was used to secure the smartphone to their lower back (Figure 2). The model of each smartphone could not be ascertained due to participants using their own phones.

**Figure 3:**
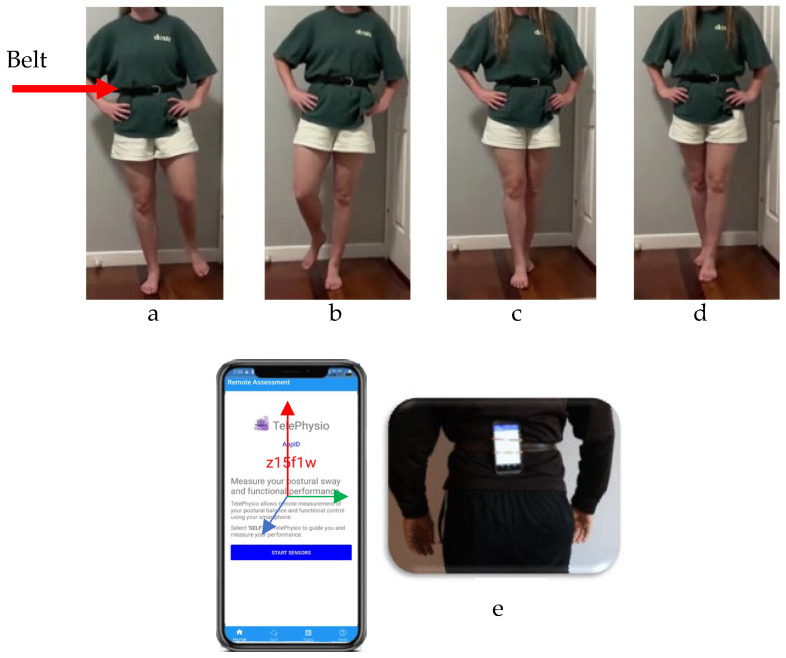
Balance tests performed in this study: single-leg stance (**a**,**b**) and tandem stance (**c**,**d**), with the participants’ eyes closed (both left and right legs), on a hard surface. The smartphone was placed at the lower back attached to a belt with the smartphone screen facing away from the body in a portrait layout, with the top side facing upwards (**e**). The sensor axes are illustrated with red (*y*-axis), green (*x*-axis), and blue (*z*-axis).

Balance tasks comprised single-leg and tandem stance with the participants’ eyes closed for both left and right legs on a hard surface (see Figure 3). Each balance trial lasted 22.5 s and began with the command “START” from the smartphone when the researcher pressed the start button in their browser interface. This initiated the smartphone motion-sensor data capture. Once the balance trial started, the researcher visually observed the participant and scored their performance using the modified BESS test scoring criteria. At the end of each balance trial, the researcher recorded the modified BESS score from their observation and checked the smartphone data that was automatically uploaded to the cloud. All balance trials and BESS test scoring were conducted by the same physiotherapist with 5 years of experience completing balance tests in a private outpatient physiotherapy clinic.

### 2.4. Data Analysis

For each balance trial, a modified BESS score was given by counting the errors or deviations from the proper stance accumulated by the participant. A single point was given each time a participant made the following error: (1) lifting hands off the iliac crest; (2) opening eyes; (3) stepping, stumbling, or falling; (4) moving the hip into >30 degrees of abduction; (5) lifting the forefoot or heel; and (6) remaining out of test position for >5 s. The maximum total number of errors for any single trial was 11. The modified BESS test data were measured as a continuous variable only, and no pre-designated thresholds (i.e., low, moderate, high) were used to classify participants into a certain group. All acceleration data from the smartphone motion sensors were processed by eliminating the gravity component by subtracting the acceleration signal mean, followed by a zero-phase Butterworth high-pass filter at 0.3 Hz, and then a third-order Savitzky–Golay smoothing filter with frames of 41 points [34]. Postural sway was then evaluated by calculating the (1) average acceleration magnitude from the mean for the mediolateral and anterior–posterior directions for both single-leg and tandem stance (SLMagML, SLMagAP, TanMagML, TanMagAP, respectively), and (2) the root-mean-square acceleration for mediolateral and anterior–posterior directions (SLrmsML, SLrmsAP, TanrmsML, and TanrmsAP, respectively) for each of the balance trials [34].

### 2.5. Statistical Analysis

Due to the exploratory nature of this cross-sectional study, which did not carry a pre-defined hypothesis, a formal sample size calculation was not needed [35]. All analyses were performed using the open-source statistical software R version 4.2.1 (https://www.r-project.org/ (accessed on 8 July 2022)) with the significance level set at 0.05. An Analysis of Variance (ANOVA) was used to determine between-group differences in height, body mass, BMI, and iHOT-33 scores between symptomatic and asymptomatic male and female groups. A Tukey post hoc analysis test was performed to identify between-group differences.

Exploratory Factor Analysis (EFA) was used to explore the discriminative validity of the smartphone sensor system in contrast with the use of the modified BESS test to detect changes in static balance measures. Due to the exploratory nature of the EFA, the factor loadings and analysis did not involve a hypothesised a priori. The EFA was used over other statistical methods [36] to (1) establish prior unknown relationships between factors and (2) explore the number of factors into which the data could be loaded [36,37]. The EFA aimed to subsequently explain the overall balance data obtained from both the smartphone and modified BESS test to find patterns and relationships by helping to reduce dimensionality and placing each variable within common factors (constructs). The values for uniqueness, communality, and complexity were also calculated as part of the EFA. A factor explains both the relationships and the amount of variance that is associated across several different variables [38]. Uniqueness refers to how much a variable is unable to be explained by factors; communality refers to how much a variable can be explained by a factor [37,39].

Each variable from the smartphone and modified BESS test was then input into a Clustered Receiver Operating Characteristic (ROC) analysis to evaluate whether these measures could effectively discriminate between participants with and without hip pain. A clustered ROC was used, due to its capacity to handle multiple clustered (i.e., repeated measures) data points (i.e., two hips) from the same participant [40]. The Area Under the Curve (AUC) was used to summarise the ROC, with values of 0.5–0.7 deemed no-to-poor, 0.7–0.8 acceptable, 0.8–0.9 excellent, and >0.9 outstanding discriminative ability [41]. Sensitivity, specificity, and confidence intervals were reported alongside the AUC.

The smartphone data was used to assess for any significant differences between biological sex (male or female) and hip pain status (symptomatic or asymptomatic hip) for each smartphone variable. A Shapiro–Wilk normality test was used to test the variance for each balance smartphone variable. A log transformation was then applied to any significantly skewed data. A Linear Mixed-Effects Analysis of Covariance (ANCOVA), with a previous ankle injury as the covariate, was undertaken to assess for any significant differences between biological sex and hip pain status. This approach was taken due to the ability of a Linear Mixed-Effects ANCOVA to analyse data as non-independent (i.e., random effects), given that each participant had two separate hips. A previous ankle injury was used as a covariate to determine whether factors other than the hip affected balance measures in this cohort. Data were reported as a *p*-value, an F-statistic with its associated degrees of freedom (df), a marginal R^2^, and an effect size using a partial eta squared (ηp^2^) and a fixed effects estimate (β) per hip with confidence intervals (CI). The effect sizes, using the partial eta squared (ηp^2^), were based on the original Cohen’s d [42] and interpreted as small (≤0.01), medium (≤0.06), and large (≥0.14) [43,44]. A Tukey post hoc analysis test was performed to identify specific between-group differences.

## 3. Results

Baseline characteristics, which included age (years) (range: 18–40), height (m) (range: 1.55–1.94), BMI (kg/m^2^) (range: 18.98–49.73), body mass (kg) (range: 57–122.6) and iHOT-33 (range: 50.62–100) values are outlined in Table 1. These are represented as a mean (SD) for both symptomatic and asymptomatic male and female groups. The post hoc analysis showed that males were significantly taller than females (*p* < 0.001), symptomatic females had a significantly greater BMI compared to asymptomatic and symptomatic males (*p* < 0.002), and asymptomatic males’ iHOT-33 scores were significantly greater than symptomatic females’ scores (*p* = 0.021).

### 3.1. Exploratory Factor Analysis

The parallel analysis demonstrated that the extraction of two factors was appropriate to explain the balance performance variables. The EFA was undertaken to compare whether single-leg and tandem variables from either the modified BESS test or the smartphone could explain the results from each specific balance task. The EFAs are presented in Table 2 and Table 3. For the single-leg balance trials, the EFA showed that the smartphone data variables are explained by the first factor, demonstrating 0.857, 0.903, 0.891, and 0.802 for SLMagML, SLMagAP, SLrmsML, and SLrmsAP, respectively. Both the BESS test SL (0.909) and the overall BESS test score (0.812) were explained by factor two. The second EFA (Table 3) found that TanMagML (0.934), TanMagAP (0.863), TanrmsML (0.927), and TanrmsAP (0.863) were all explained by factor one. Both the BESS test tandem (0.795) and the overall BESS test score (0.557) were explained by factor two.

### 3.2. Hip Pain, Clustered Receiver Operating Characteristic

**Figure 4:**
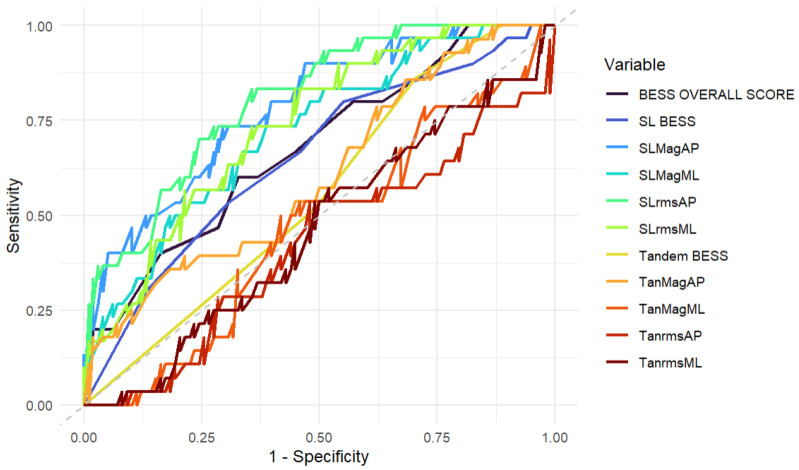
ROC and AUC. Clustered Receiver Operating Characteristic (ROC) and Area Under the Curve (AUC) predictors for the smartphone and the modified BESS score balance tests.

The variables for each outcome were input into a clustered ROC analysis (Table 4) and measured using an AUC (Figure 4). Scores ranged between 0.462 and 0.804 across all 11 variables. All single-leg balance tests in the anterior–posterior and mediolateral directions demonstrated an acceptable to excellent (AUC: 0.721–0.804) ability to discriminate and detect whether an individual had hip-related pain. All other measures demonstrated a no-to-poor ability to discriminate between symptomatic and asymptomatic individuals.

### 3.3. Between-Group Comparison

The Shapiro–Wilk normality test showed a non-normal distribution (*p* < 0.05) for all smartphone variables. A logarithmic transformation was subsequently applied to all skewed data. The results from the Linear Mixed-Effects ANCOVA showed significant differences in single-leg balance in the anterior–posterior and mediolateral directions (*p* < 0.05) (Table 5). Post hoc Tukey HSD test demonstrated that individuals with hip-related pain had a significantly lower magnitude and variability across all single-leg (MLMag: *p* = 0.048, CI: 0.000–0.086; MLrms: *p* = 0.040, CI: 0.003–0.139; APMag: *p* = 0.033, CI: 0.003–0.076; APrms: *p* = 0.026, CI: 0.010–0.166) balance variables. There were no significant differences when examining the interactions between pain and biological sex (*p* > 0.05) (Table 5). A previous ankle injury had no significant effect on any balance variable (*p* > 0.05) (Table 5).

## 4. Discussion

Smartphone sensor technology is a novel method that can be utilised by healthcare professionals, in combination with a modified BESS test, to assess static balance in individuals with musculoskeletal pain. This study compared the use of smartphone technology and the modified BESS test method to both discriminate and explore the differences in balance metrics between soccer athletes with and without hip-related pain. The results from the EFA demonstrated that the two different balance assessments independently explain balance performance, due to their loading onto separate latent factors, thereby measuring a different set of balance constructs. For example, within the EFA measuring single-leg balance, factor one was loaded quite highly by the smartphone (0.802–0.903), and to a much lesser extent, by the single-leg BESS (0.273) and by overall BESS tests (0.275). On the other hand, factor two was loaded highly by both BESS tests (0.812–0.909) and, again, to a much lesser extent by the smartphone device variables (0.186–0.367). These results suggest that despite the small overlap between measurements, these two balance tests provide a different set of balance information for clinicians. This is important in a clinical context, where the modified BESS test can be used to detect errors (i.e., abduct the hip > 30°), and the smartphone can measure continuous and subtle changes in trunk sway. Including the smartphone within a balance assessment testing battery can provide additional value to what a regular clinical visual assessment may not fully capture. For example, the limitation of the modified BESS test is its subjective score, calculated through the number of errors an individual makes during a certain balance task. While errors in the BESS test reflect a change in movement (i.e., abduct the hip > 30°), its scoring system is unable to provide quantitative data on directional movement—such as small changes in the anterior–posterior and mediolateral direction—to clinicians, which may limit the understanding of a balance impairment in individuals with hip-related pain. While visual assessments have in the past been recommended to assess static balance [10], the study demonstrated that the smartphone application can provide clinicians with extra information for a standard modified BESS test. Integrating smartphone sensor systems into clinical assessments may therefore bridge the gap between the limitations seen in a modified BESS test by providing a more detailed quantification of static balance performance. This, coupled with previous research demonstrating the validity of smartphone sensors compared to gold standard instruments [27,30], suggests that clinicians should integrate smartphone devices into a clinical assessment to eventually guide more specific treatment plans for their patients.

This is the first study to analyse the discriminative ability of a smartphone application and a modified BESS test to differentiate between those with and without hip-related pain. Overall, both the modified BESS test and the smartphone application measuring tandem stance performed poorly in discriminating between whether an individual had hip-related pain. The smartphone’s ability to measure the magnitude and variability in the anterior–posterior and mediolateral directions during single-leg stance, on the other hand, demonstrated acceptable-to-excellent discriminative validity (AUC: 0.721–0.804). Despite this, it must be acknowledged that the lower bound confidence intervals for all single-leg variables (SLMagAP, SLMagML, and SLrmsML) fell below the 0.70–0.80 acceptable AUC threshold. This implies that the smartphone device may incorrectly deem an individual to be either symptomatic or asymptomatic of hip-related pain when measuring single-leg balance, and caution should be applied when interpreting the results.

All single-leg balance variables also showed a lower overall specificity (0.551–0.704) compared to their sensitivity (0.733–0.833). This indicates that although the smartphone can identify most individuals with hip-related pain (sensitivity), it has a lower ability to detect individuals who do not have hip pain. The device may therefore produce a false-positive by incorrectly deeming an individual to have hip-related pain when they do not. False-positive results stemming from the smartphone device may be due to several different factors. Firstly, the nature of a remote assessment may have reduced the researcher’s ability to confirm the central position of the smartphone on the participant’s lower back and altered the feedback received from the phone’s internal gyroscope and accelerometer systems. Secondly, although the smartphone was attached to a belt, a sudden loss of balance and rapid change in trunk position during testing may have permanently changed the fixation of the phone for the remainder of the test, altering the results. Finally, heterogeneity between different smartphone devices may have provided slightly different data across participants. For these reasons, researchers recommend that clinicians ensure that the phone is attached securely to a patient’s belt prior to testing (i.e., through a pre-testing check) and maintain consistency in the testing protocols used, especially if repeating a test over time. When testing remotely, it is recommended to include video conference interaction, such as Microsoft Teams, so that the clinician can view the smartphone position and patient posture during testing.

Poor discriminative ability in the BESS test and smartphone technology measuring tandem stance may not particularly reflect a shortcoming of the smartphone motion sensors or the modified BESS test, but rather the potentially inappropriate use of some static balance tests for individuals with hip-related pain. The equivalencies in most measurement methods for the BESS and smartphone sensor variables may instead indicate that static balance in soccer athletes with hip-related pain is not a challenging and sensitive assessment. While previous studies have shown the ability of the smartphone sensor systems to discriminate between static balance impairments in individuals with and without concussion [23], ankle instability [29], and stroke [26] in the non-athletic populations, athletes may require more challenging dynamic tasks to detect changes in postural sway. Therefore, future studies should focus on postural balance impairments in more challenging dynamic balance tasks, particularly in soccer athletes with hip-related pain.

In line with the second aim of this study, it was demonstrated that individuals with hip-related pain had a lower magnitude and variability of trunk postural sway in both the anterior–posterior and mediolateral directions during single-leg stance, compared to individuals without hip-related pain. These results may be explained by the notion that symptomatic individuals have less movement variation and use fewer sensory-motor options when controlling their trunk during single-leg stance. Removing the eyes as a source of visual stimuli (i.e., completing static balance tasks with eyes closed) increases the reliance on the interactions between the vestibular and proprioceptive (i.e., Golgi tendon organ, joint mechanoreceptors, and muscle spindle) systems working in a dynamic feedforward and feedback control couple [45,46]. Previous research in non-hip-related conditions, such as chronic lower back pain [47], patella tendinopathy [48], and CAI [49], has shown lower movement variability in these populations [50], potentially as a way to protect a certain nociceptive tissue or structure [51]. This may be due to the impaired motor control pathways in both the proprioceptive and central processing systems that are present in individuals with pain [46]. In agreement with these results, one study by Marshall et al. (2023) found that individuals with femoroacetabular impingement syndrome, a component of hip-related pain [52], had reduced trunk sway in the mediolateral direction when completing a double-leg squat movement, compared to healthy individuals, when assessed using smartphone sensor systems [27]. This finding represents a similarity between the static balance results found within this study and the lower dynamic balance movement variability found in other participants with hip pain [27].

This finding holds clinical relevance for health practitioners who treat athletes with hip-related pain. It is suggested that clinicians integrate the smartphone platform when assessing static single-leg balance in both the anterior–posterior and mediolateral directions. Balance impairments that are found in these individuals may then be used to guide treatment. Whilst static balance testing in individuals with hip-related pain may hold somewhat limited merit, and would only be appropriate to assess single-leg stance in athletes with hip-related pain, other dynamic tests using the smartphone may be more clinically useful due to previously reported balance differences during functional dynamic tasks [27,53]. Although previous research [19,28] has reported these differences, studies investigating the sensitivity of sensor-based applications compared to clinical outcome measures [54] have only explored ambulation as a dynamic functional task in populations with a neurological disease [54,55]. Despite these measures being able to discriminate between neurological conditions and displaying superiority to traditional walking tests [54], there have been no studies that examine more demanding dynamic balance movements in a sporting population. Future research should therefore prioritise investigating dynamic sports-specific tasks such as unilateral and bilateral jumping or change in direction movements, which may hold more merit for athletes experiencing hip-related pain. IN the future, the use of smartphone devices may have the potential to provide more evidence for hip-related conditions being a dynamic entity.

### Limitations

It is acknowledged that some limitations in this study exist. Both the modest sample size (n = 64) and size differences between asymptomatic males and all other groups may have reduced the strength of the Linear Mixed-Effects ANCOVA, especially when considering the interactions between biological sex and hip-related pain. For example, there was a large size discrepancy between asymptomatic females (n = 10, 20 hips), symptomatic males (n = 7, 14 hips), symptomatic females (n = 8, 16 hips), and asymptomatic males (n = 39, 78 hips) within each group. This may have increased the likelihood of a Type I error and led to an overall reduction in the statistical power and generalizability of these findings. Furthermore, the testing protocol did not attain a pain score during each balance test trial. Understanding whether a trial caused hip pain could allow researchers to ascertain whether differing results were due to the onset of symptoms and should be implemented within future research.

Testing static balance is not fully representative of the demands and/or movements seen within soccer training and games. For many athletes, hip-related pain may be only provoked during dynamic movement, and there is the potential for static balance tests to not fully reproduce movements that cause symptoms. This can also be extended to Femoroacetabular Impingement Syndrome, a subset of hip-related pain [52], in which pain originates from the combined internal rotation and flexion of the hip [56], and may be better reproduced during dynamic movement [57]. Further research should therefore examine dynamic balance movements, which are specific to soccer and pertain to tasks that may be more provocative of symptoms. Furthermore, while a FADIR test was used to decipher the potential origin of pain (i.e., intracapsular or extracapsular), the inclusion of individuals with hip-related pain was self-reported by the participant. This is a limitation within our study, as self-reporting measures may have introduced heterogeneity in participants with hip-related pain and affected the validity of the results.

It is acknowledged that a variety of different smartphone models were used for each participant. Although this allows individuals to be assessed with their own device online, which is advantageous when assessing individuals remotely, each model contains its own accelerometer and gyroscope motion sensors. Within this study, the operating system, model, brand, and year of each participant’s smartphone were not recorded, and the possible influence of differing models on data recording was not evaluated. This heterogeneity between participant devices represents a major limitation within the study, due to the potentially different quality of sensors and sampling frequencies within each device, which reduces the validity and reproducibility of the results. Despite this, previous works by Marshall et al. (2022, 2023) have shown very good validity and reliability across a range of iPhone (iPhone 8, iPhone 8 plus, iPhone SE 2020, X, SE, 11, 12, and 13) and Android (Samsung Galaxy, Huawei P10, Google pixel 6, Samsung Galaxy s20fe, Samsung Galaxy A8, and Xiaomi POCO X3 NFC) models [27,58].

## 5. Conclusions

This study recommends the use of smartphones in postural balance assessment. The modified BESS and smartphone balance tests independently explain balance performance. The smartphone device has the capability of discriminating between individuals with and without hip-related pain when assessing single-leg static balance tasks. Individuals with hip-related pain displayed less variation in the anterior–posterior and mediolateral planes during single-leg balance tests compared to healthy individuals, which suggests an altered motor variability in this cohort. It is suggested that future cross-sectional or longitudinal studies explore dynamic balance assessments to identify athletes with hip pain.

## Figures and Tables

**Table 1:** Means and standard deviations of participants’ characteristics and reporting outcome measure iHOT-33 for each group.

Group	Number	Age	Height	Mass	BMI	iHOT-33
Asymptomatic Females	n = 10 (20 hips)	24.20 (5.51)	1.67 (0.08)	69.70 (10.03)	25.26 (4.89)	84.96 (14.42)
Asymptomatic Males	n = 39 (78 hips)	22.38 (4.70)	1.80 (0.07)	77.04 (11.51)	23.76 (2.74)	87.12 (10.25)
Symptomatic Females	n = 8 (16 hips)	27.00 (5.95)	1.64 (0.09)	80.45 (22.96)	30.51 (10.82)	73.29 (14.00)
Symptomatic Males	n = 7 (14 hips)	21.43 (3.74)	1.77 (0.07)	72.71 (11.50)	23.10 (2.40)	83.87 (14.52)

**Table 2:** Exploratory Factor Analysis for single-leg trials. SLMagML: single-leg mediolateral; SLMagAP: single-leg anterior–posterior; SLrmsML: single-leg variability mediolateral; SLrmsAP: single-leg variability anterior–posterior.

Variable	Factor 1	Factor 2	Uniqueness	Communality	Complexity
SLMagML	0.857	0.367	0.131	0.869	1.355
SLMagAP	0.903	0.247	0.123	0.877	1.149
SLrmsML	0.891	0.284	0.126	0.874	1.201
SLrmsAP	0.802	0.186	0.322	0.678	1.107
BESS SL	0.273	0.909	0.100	0.900	1.178
BESS Overall	0.275	0.812	0.266	0.734	1.227

Note: [37,38,39]. Factor explains both the relationships and the amount of variance that is associated across several different variables. Uniqueness is the degree to which a variable is not explained by factors. Communality is the extent to which a variable can be explained by factors.

**Table 3:** Exploratory Factor Analysis for the tandem stance trials. TanMagML: tandem magnitude mediolateral; TanMagAP: tandem magnitude anterior–posterior; TanrmsML: tandem variability mediolateral; TanrmsAP: tandem variability anterior–posterior.

Variable	Factor 1	Factor 2	Uniqueness	Communality	Complexity
TanMagML	0.934	0.228	0.075	0.925	1.119
TanMagAP	0.863	0.385	0.106	0.894	1.383
TanrmsML	0.927	0.267	0.070	0.930	1.165
TanrmsAP	0.863	0.407	0.089	0.911	1.424
BESS Tandem	0.375	0.795	0.227	0.773	1.425
BESS Overall	0.145	0.557	0.669	0.331	1.135

Note: [37,38,39]. Factor explains both the relationships and the amount of variance that is associated across several different variables. Uniqueness is the degree to which a variable is not explained by factors. Communality is the extent to which a variable can be explained by factors.

**Table 4:** AUC scores for all BESS tests and smartphone variables.

Variable	AUC	Confidence Interval (Low)	Confidence Interval (High)	Sensitivity	Specificity	Rating
BESS Score	0.676	0.567	0.785	0.600	0.673	No-to-poor
Tandem BESS	0.557	0.457	0.657	0.862	0.295	No-to-poor
Single-Leg BESS	0.652	0.540	0.764	0.800	0.448	No-to-poor
SLMagML	0.721	0.619	0.823	0.733	0.612	Acceptable
SLMagAP	0.781	0.689	0.874	0.733	0.704	Acceptable
SLrmsML	0.737	0.642	0.832	0.833	0.551	Acceptable
SLrmsAP	0.804	0.721	0.887	0.833	0.642	Excellent
TanMagML	0.465	0.347	0.584	0.500	0.581	No-to-poor
TanMagAP	0.607	0.488	0.726	0.392	0.785	No-to-poor
TanrmsML	0.462	0.343	0.581	0.571	0.479	No-to-poor
TanrmsAP	0.424	0.300	0.548	0.535	0.520	No-to-poor

**Table 5:** Linear Mixed-Effects ANCOVA showing significant differences in single-leg balance between individuals with and without hip-related pain. The previous ankle injury was selected as the covariance variable. Significant (<0.05) differences are presented in bold.

Variable	Biological Sex	Hip Pain	Interaction (Sex * Pain)	Previous Ankle Injury
SLMagML	*p* = 0.831 F = (1, 49.05) 0.045 R^2^: 0.137 ηp^2^: 0.000 β: 0.031 (CI: −0.019–0.081)	* *p* = 0.048 F = (1, 48.45) 4.108 R^2^: 0.137 ηp^2^: 0.078 (medium) β: −0.016 (CI: 0.080–0.046)	*p* = 0.222 F = (1, 48.27) 1.529 R^2^: 0.137 ηp^2^: 0.030 β: −0.052 (CI: −0.138–0.033)	*p* = 0.834 F = (2, 47.73) 0.181 R^2^: 0.137 ηp^2^: 0.007 β: 0.008 (CI: −0.022–0.039)
SLMagAP	*p* = 0.339 F = (1, 50.97) 0.930 R^2^: 0.214 ηp^2^: 0.017 β: 0.045 (CI: 0.002–0.088)	* *p* = 0.033 F = (1, 50.56) 4.781 R^2^: 0.214 ηp^2^: 0.086 (medium) β: −0.011 (CI: −0.06–0.042)	*p* = 0.127 F = (1, 50.44) 2.397 R^2^: 0.214 ηp^2^: 0.045 β: −0.056 (CI: −0.129–0.016)	*p* = 0.690 F = (2, 55.63) 0.347 R^2^: 0.214 ηp^2^: 0.012 β: 0.008 (CI: −0.019–0.035)
SLrmsML	*p* = 0.724 F = (1, 50.49) 0.125 R^2^: 0.157 ηp^2^: 0.002 β: 0.054 (CI: −0.025–0.133)	* *p* = 0.040 F = (1, 49.91) 4.420 R^2^: 0.157 ηp^2^: 0.081 (medium) β: −0.028 (CI: −0.128–0.070)	*p* = 0.217 F = (1, 49.74) 1.558 R^2^: 0.157 ηp^2^: 0.030 β: −0.084 (CI: −0.219–0.078)	*p* = 0.690 F = (2, 48.93) 0.372 R^2^: 0.157 ηp^2^: 0.015 β: 0.012 (CI: −0.035–0.060)
SLrmsAP	*p* = 0.654 F = (1, 55.30) 0.201 R^2^: 0.149 ηp^2^: 0.003 β: 0.046 (CI: −0.046–0.139)	* *p* = 0.025 F = (1, 55.47) 5.280 R^2^: 0.149 ηp^2^: 0.086 (medium) β: −0.059 (CI: −0.173–0.054)	*p* = 0.456 F = (1, 55.74) 0.562 R^2^: 0.149 ηp^2^: 0.009 β: −0.057 (CI: −0.212–0.096)	*p* = 0.613 F = (2, 66.70) 0.491 R^2^: 0.149 ηp^2^: 0.014 β: 0.023 (CI: −0.043–0.089)
TanMagML	*p* = 0.818 F = (1, 42.86) 0.053 R^2^: 0.030 ηp^2^: 0.001 β: −0.013 (CI: −0.056–0.028)	*p* = 0.329 F = (1, 42.15) 0.972 R^2^: 0.030 ηp^2^: 0.022 β: −0.027 (CI: −0.081–0.025)	*p* = 0.616 F = (1, 42.03) 0.254 R^2^: 0.030 ηp^2^: 0.006 β: 0.018 (CI: −0.056–0.094)	*p* = 0.602 F = (2, 44.95) 0.512 R^2^: 0.030 ηp^2^: 0.022 β: 0.009 (CI: −0.018–0.037)
TanMagAP	*p* = 0.825 F = (1, 38.04) 0.049 R^2^: 0.081 ηp^2^: 0.001 β: −0.005 (CI: −0.038–0.028)	*p* = 0.084 F = (1, 37.66) 3.143 R^2^: 0.081 ηp^2^: 0.077 β: −0.034 (CI: −0.076–0.007)	*p* = 0.566 F = (1, 37.69) 0.334 R^2^: 0.081 ηp^2^: 0.008 β: 0.016 (CI: −0.042–0.076)	*p* = 0.517 F = (1, 52.24) 0.666 R^2^: 0.081 ηp^2^: 0.024 β: 0.011 (CI: −0.012–0.034)
TanrmsML	*p* = 0.689 F = (1, 43.58) 0.162 R^2^: 0.032 ηp^2^: 0.003 β: −0.022 (CI: −0.087–0.042)	*p* = 0.363 F = (1, 43.17) 0.844 R^2^: 0.032 ηp^2^: 0.019 β: −0.037 (CI: −0.117–0.043)	*p* = 0.694 F = (1, 43.16) 0.156 R^2^: 0.032 ηp^2^: 0.003 β: 0.022 (CI: −0.091–0.136)	*p* = 0.523 F = (2, 54.23) 0.655 R^2^: 0.032 ηp^2^: 0.023 β: 0.025 (CI: 0.019–0.069)
TanrmsAP	*p* = 0.790 F = (1, 41.36) 0.071 R^2^: 0.084 ηp^2^: 0.001 β: −0.008 (CI: −0.067–0.049)	*p* = 0.129 F = (1, 41.78) 2.396 R^2^: 0.084 ηp^2^: 0.054 β: −0.054 (CI: −0.125–0.016)	*p* = 0.535 F = (1, 42.53) 0.390 R^2^: 0.084 ηp^2^: 0.009 β: 0.031 (CI: −0.070–0.132)	*p* = 0.303 F = (1, 64.31) 1.213 R^2^: 0.084 ηp^2^: 0.036 β: 0.031 (CI: 0.010–0.074)

Note: * significant *p*-value; F = F-statistic (df); df = degrees of freedom; R^2^ = marginal R^2^; ηp^2^ = partial eta squared (small ≤ 0.01, medium ≤ 0.06, and large ≥ 0.14); fixed effects estimate (β) with CI.

## Data Availability

The datasets presented in this article are not readily available because of ethical restrictions.
